# Influence of Planets

**Published:** 1888-04

**Authors:** G. W. Hunter


### INFLUENCE OF PLANETS.

Richard A. Proctor, the astronomer, writing on astrology, says :—"We are apt to speak of astrology as though it were an altogether contemptible superstition, and to contemplate with pity those who believed in it in old times. . . . . . Indeed, all other methods of divination of which I have ever heard, are not worthy to be mentioned in company with astrology, which, if a delusion, has yet had a foundation in thoughts well worthy of consideration. The heavenly bodies *do* rule the fates of men and nations in the most unmistakable manner, seeing that without the controlling and beneficent influences of the chief among those orbs—the sun—every living thing on the earth must perish."

Speaking of the influence of the moon on the tides of the ocean, he remarks, "Seeing that two of the orbs are thus influencing our earth, is it not natural that the other moving bodies should be thought to possess also their special power?

Scientists suggested long ago that from the sun come coloring rays, heating rays and actinic, or chemical rays ; that each of these produces its own respective line in the spectrum, and that each travels according to a velocity of its own, varying from 458 millions of millions per second for the extreme red ray, to 727 millions of millions for the extreme violet. It is said that sun light will kill some kinds of minute animal life, and that "sun-spots" have some influence on the weather, and, may be, cause potato rot.

Every school-boy knows something of the magnetic power the sun possesses in its attraction of the planets. Yet how little does science know what secret power is hid behind this "attraction"—this spiritual influence which draws all things. There is a line of communication between us and all the visible planets, at least, because light has found the path and comes to tell us that "they are." When we know what light is, in its effects, we will know some things of the message it brings from yonder star, far away on the border of a shoreless space.

By the rules of scientists, every atom in my body is in sympathy,—by the law of attraction,—with every atom of this great universe. I am, therefore, in attractive communion with all my surroundings and the product of multiplied millions of millions of factors, all of which are in the heavens.

The ancients knew that planets affected man, and by some power akin to "natural survival," all well-to-do almanacs still have the signs girdling the man, each throwing its ray of influence to some part of his anatomy. This pictorial frontispiece of your almanac is a relic of this ancient science.—G. W. HUNTER, *in The Esoteric.*

